# Trigger factors in patients with a patent foramen ovale—associated stroke: A case-crossover study

**DOI:** 10.1177/17474930241242625

**Published:** 2024-04-02

**Authors:** Maikel HM Immens, Merel S Ekker, Esmee Verburgt, Jamie I Verhoeven, Mijntje MI Schellekens, Nina A Hilkens, Esther M Boot, Mayte E Van Alebeek, Paul JAM Brouwers, Renate M Arntz, Gert W Van Dijk, Rob AR Gons, Inge WM Van Uden, Tom den Heijer, Paul LM de Kort, KF de Laat, Anouk GW Van Norden, Sarah E Vermeer, Marian SG Van Zagten, Robert J Van Oostenbrugge, Marieke JH Wermer, Paul J Nederkoorn, Henk Kerkhoff, FA Rooyer, Frank G Van Rooij, Ido R Van den Wijngaard, Catharina JM Klijn, Anil M Tuladhar, Tim JF ten Cate, Frank-Erik de Leeuw

**Affiliations:** 1 Department of Neurology, Donders Institute for Brain, Cognition, and Behaviour, Radboud University Medical Center, Nijmegen, The Netherlands; 2 Department of Neurology, Amphia Hospital, Breda, The Netherlands; 3 Department of Neurology, Medisch Spectrum Twente, Enschede, The Netherlands; 4 Department of Neurology, Canisius Wilhelmina Hospital, Nijmegen, The Netherlands; 5 Department of Neurology, Catharina Hospital, Eindhoven, The Netherlands; 6 Department of Neurology, Franciscus Gasthuis & Vlietland, Rotterdam, The Netherlands; 7 Department of Neurology, Elisabeth-TweeSteden Hospital, Tilburg, The Netherlands; 8 Department of Neurology, Haga Hospital, The Hague, The Netherlands; 9 Department of Neurology, Rijnstate Hospital, Arnhem, The Netherlands; 10 Department of Neurology, Jeroen Bosch Hospital, ‘s-Hertogenbosch, The Netherlands; 11 Department of Neurology, Maastricht University Medical Center+, Maastricht, The Netherlands; 12 Department of Neurology, Leiden University Medical Center, Leiden, The Netherlands; 13 Department of Neurology, Amsterdam University Medical Center, Location AMC, Amsterdam, The Netherlands; 14 Department of Neurology, Albert Schweitzer Hospital, Dordrecht, The Netherlands; 15 Department of Neurology, Zuyderland Hospital, Sittard-Geleen, The Netherlands; 16 Department of Neurology, Medical Center Leeuwarden, Leeuwarden, The Netherlands; 17 Department of Neurology, Haaglanden Medical Center, The Hague, The Netherlands; 18 Department of Cardiology, Radboud University Medical Center, Nijmegen, The Netherlands

**Keywords:** Patent foramen ovale, young stroke, trigger factors

## Abstract

**Background:**

Patent foramen ovale (PFO) is a congenital anatomical variant which is associated with strokes in young adults. Contrary to vascular risk factors and atherosclerosis, a PFO is present from birth. However, it is completely unknown how an anatomical structure that is already present at birth in a large proportion of the population can convert into a PFO that causes stroke in a few. Recent studies reported a significant association between certain trigger factors and ischemic stroke in young adults. This study aims to investigate these triggers in PFO-associated stroke.

**Methods:**

The ODYSSEY study, a multicenter prospective cohort study between 2013 and 2021, included patients aged 18–49 years experiencing their first-ever ischemic event. Participants completed a questionnaire about exposure to potential trigger factors. A case-crossover design was used to assess the relative risks (RR) with 95% confidence intervals (95% CI). The primary outcome was the RR of potential trigger factors for PFO-associated stroke.

**Results:**

Overall, 1043 patients completed the questionnaire and had an ischemic stroke, of which 124 patients had a PFO-associated stroke (median age 42.1 years, 45.2% men). For patients with PFO-associated stroke, the RR was 26.0 (95% CI 8.0–128.2) for fever, 24.2 (95% CI 8.5–68.7) for flu-like disease, and 3.31 (95% CI 2.2–5.1) for vigorous exercise.

**Conclusion:**

In conclusion, flu-like disease, fever, and vigorous exercise may convert an asymptomatic PFO into a stroke-causing PFO in young adults.

**Data access statement:**

The raw and anonymized data used in this study can be made available to other researchers on request. Written proposals can be addressed to the corresponding author and will be assessed by the ODYSSEY investigators for appropriateness of use, and a data sharing agreement in accordance with Dutch regulations will be put in place before data are shared.

## Introduction

The foramen ovale allows for right-to-left shunting pre-birth and usually spontaneously closes after birth. However, in about 25% of individuals, it remains patent.^1^ This patent foramen ovale (PFO) is a possible cause of ischemic stroke in selected patients, without any other cause of stroke. PFO closure is proven beneficial in these selected patients over medical therapy in preventing recurrent ischemic stroke.^2,3^ Given the high prevalence of PFO, it will most often be an innocent bystander, as the majority of patients with a stroke, also those with a PFO, will have another cause of stroke.

Contrary to vascular risk factors and atherosclerosis, a PFO is present from birth. However, it is completely unknown how an anatomical structure that is already present at birth in a large proportion of the population can convert into a PFO that causes stroke in a few. The relatively new concept of trigger factors may elucidate this pathological conversion. A trigger factor is a short-lasting exposure to a trigger (i.e. toxins, caffeine, sexual activity, physical exercise, or infection), that may subsequently create a (short-lasting) condition (e.g. a prothrombotic state or increase in blood pressure) that may predispose to stroke.^4–6^ Recent studies reported a significant association between trigger factors (cola consumption, vigorous physical exercise, sexual activity, illicit drug use, fever and flu-like disease) and ischemic stroke in young adults (< 50 years).^7,8^ To date, only one, case series (*n* = 4) investigated the relation between a trigger factor (exercise-induced Valsalva) and PFO-associated stroke.^9,10^ In practice, only a small percentage of patients who experience a PFO-associated stroke reportedly performed a Valsalva-like maneuver prior to the event. Besides, more than 50% of patients with a PFO have permanent right-to-left shunting, also without a Valsalva-like maneuver.^11^

There is evidence suggesting that a PFO, unlike atrial fibrillation or atherosclerotic plaques, is not a direct cause of stroke; instead, it facilitates the passage of a venous thrombus into the arterial circulation.^12^ We hypothesize, therefore, that other trigger factors create environmental conditions leading to a prothrombotic environment, causing a PFO-associated stroke. Hence, we investigated the relationship between potential trigger factors (caffein-containing coffee consumption, caffein-containing cola consumption, alcohol consumption, cigarette smoking, illicit drug use, vigorous physical exercise, sexual activity, fever and flu-like disease) and the risk of stroke at young age in patients with a PFO-associated stroke.

## Methods—study population

We performed a case-crossover study as part of the Observational Dutch Young Symptomatic StrokE studY (ODYSSEY), a multicenter prospective cohort study on the prognosis and risk factors of patients aged 18–49 years with a first-ever ischemic stroke or intracerebral hemorrhage (ICH).^13^ Ischemic stroke was defined according to a tissue-based definition as acute onset of a neurologic deficit with imaging proof of ischemia. We included patients between May 2013 and February 2021. Exclusion criteria were any type of subarachnoid hemorrhage (SAH), cerebral venous sinus thrombosis, or a history of a clinically symptomatic transient ischemic attack (TIA), ischemic stroke, or ICH.

### Baseline data collection

We systematically collected information including stroke characteristics and severity (National Institutes of Health Stroke Scale, NIHSS), diagnostic laboratory and cardiac tests, (vascular) risk factors, causes of stroke according to the Trial of Org 10172 in Acute Stroke Treatment (TOAST) classification, and medication at discharge.^13,14^

### Diagnosis of a PFO and its role in stroke

Most patients underwent transthoracic echocardiography (TTE) with a bubble test using agitated saline to assess the presence of a PFO. In cases where it remained unclear whether the patient had a PFO or further evaluation was required, they underwent a transesophageal echocardiography (TEE). If the cause of stroke was evident early in the diagnostic process (e.g. carotid artery dissection, significant carotid artery stenosis), and additional information regarding the presence of PFO would be of no value, the patients would not receive an echocardiography. In June 2018, we established a multidisciplinary Heart-Stroke Team (HST) at the Radboud University Medical Center to evaluate the relationship between PFO and stroke.^15^ Patients evaluated in this multidisciplinary team underwent a thorough work-up (cardiac evaluation, prothrombosis screening, and neuroimaging). Afterward, the HST decided whether the stroke was most likely to be caused by the PFO. Prior to the HST, patients were classified by their neurologist based on the available knowledge of that time, derived from the randomized control trials (RCTs) (2016) on PFO closure (a cryptogenic stroke, below the age of 60 years and with any type of PFO).^16–18^ As the understanding of PFO-associated stroke rapidly evolved during the inclusion period of the ODYSSEY trial, because of the appearance of the major PFO closure trials^16–18^ that reported a benefit of closure in patients with no other cause of stroke, all patients who were included in our cohort before these trials were published were re-evaluated to assess whether there was a relation between the PFO and the occurrence of stroke. These patients were re-evaluated by a panel of five researchers/ medical doctors (M.H.M.I., M.S.E., N.A.H., E.V., and J.I.V.) to determine if the patients had a PFO-associated stroke. In the re-evaluation, patients were classified as having a PFO-associated stroke when they had a genuine cryptogenic stroke with no signs of other (more likely) causes of stroke after a complete work-up. This includes no lacunar infarction with signs of white matter hyperintensity and not more than one cardiovascular risk factor (e.g. hypertension, diabetes, hyperlipidemia, smoking, and obesity). All cases that were questionable were discussed with a specialized stroke neurologist (F.E.D.L.). If there was another potential (competing) cause of stroke besides the PFO, and it was more likely to be the primary cause, the patient would be classified within that category. If the cause of the stroke was attributed to another etiology (Table 1) rather than the PFO, the PFO was considered to be an innocent bystander.

**Table 1.** Baseline demographics.

Demographics	PFO-associated stroke (*n* = 124)	Bystander PFO (*n* = 48)
Men, *n* (%)	56 (45.2)	21 (43.7)
Median age (IQR)	42.1 (31.1–53.1)	43.9 (36.0–51.8)
Vascular risk factors, *n* (%)		
Smoking	35 (28.2)	30 (62.5)
Hypertension	9 (7.3)	11 (22.9)
Diabetes mellitus	5 (4.0)	4 (8.3)
Hypercholesterolemia	62 (50)	37 (77.1)
Territorial infarction, *n* (%)	109 (87.9)	40 (83.3)
Percutaneous PFO closure, *n* (%)	83 (66.9)	x
RoPE score, *n* (%)^*^		
0–5	0 (0.0)	2 (6.9)
6	7 (5.9)	3 (10.3)
7	27 (22.9)	14 (48.3)
8	50 (42.4)	8 (27.6)
9	22 (18.6)	2 (6.9)
10	12 (10.2)	0 (0.0)
Median RoPE score (IQR)	8 (6–10)	7 (6–8)
TOAST classification, *n* (%)		
Large artery atherosclerosis	x	2 (4.2)
Likely atherothrombotic stroke	x	21 (43.8)
Small vessel disease	x	6 (12.5)
Cardioembolic	x	0 (0.0)
Other determined cause	x	16 (33.3)
Multiple causes	x	1 (2.1)
Cryptogenic	x	2 (4.2)

PFO: Patent foramen ovale; IQR: interquartile range; TOAST: Trial of Org 10172 in Acute Stroke Treatment.

* Missing data on RoPE score: 6 in the “PFO-associated stroke” group, 19 in the “Bystander PFO” group.

### Assessment of trigger factors

Patients completed a structured, standardized questionnaire on exposure to potential trigger factors within predefined hazard periods (Supplemental material). Based on the previous studies, we recorded validated trigger factors, including caffein-containing coffee consumption, caffein-containing cola consumption, alcohol consumption, cigarette smoking, any illicit drug use (cocaine, heroin, methadone, amphetamine, ecstasy, and d-lysergic acid diethylamide (LSD) were considered hard drugs, cannabis products, and mushroom-containing psilocin-considered soft drugs), vigorous physical exercise, sexual activity, fever, and flu-like disease.^6,7,19–23^ Illicit drug use was combined due to the small sample size. The crossover design presumes that exposure to a trigger factor within a predefined period of time increases the risk of stroke compared to non-exposure. Patients were asked about their frequency of exposure to each trigger factor in the previous year and exposure during a predefined hazard period which was specific for each trigger factor based on its estimated duration of the trigger effect.^19–22,24–26^ Different grades of vigorous physical exercise were classified based on their metabolic equivalent of task (MET), an objective measure of the ratio of the rate at which a person expends energy, relative to the mass of that person, while performing specific physical activity compared to the resting metabolic rate. One MET is equivalent to the resting metabolic rate, for example sitting quietly, and results in burning 1 kcal/kg/h. As an example, a patient with a weight of 70 kg performing a one MET activity (sitting) for 1 h will use 70 kcal. We analyzed the following subcategories: heavy exercise [MET] = 6, severe exercise [MET] = 7, and extreme exercise [MET] = 8.

To minimize recall bias, patients were asked to complete the questionnaire as soon as possible after stroke. In the sensitivity analysis, we excluded patients who completed the questionnaire > 4 weeks after stroke (Table 3). Furthermore, unreliable answers were excluded (e.g. inconsistent answers, unmistakable false answers) before analyses were done.

## Data analyses

We examined the exposure of potential trigger factors in a hazard period compared to a control period using a case-crossover design.^5,6^ In this design, patients serve as their own control thereby minimizing the occurrence of confounding bias. Relative risk (RR) with 95% confidence interval (CI) was calculated for each potential trigger factor for ischemic stroke using the Mantel–Haenszel case-crossover method. The RR was determined by calculating the ratio of exposure in the hazard period and reported yearly exposure frequency based on patients’ weekly or daily average frequency.^5^ The RRs should be interpreted as for a short-term period and not as cumulative risks for a long-term period.

Data were reported according to the Strengthening the Reporting of Observational Studies in Epidemiology (STROBE) reporting guidelines and analyzed using SPSS Software version 22 (IBM) and R version 4.1.2 (R Project for Statistical Computing).

## Standard protocol approvals, registrations, and patient consents

The Medical Review Ethics Committee region Arnhem–Nijmegen approved the study (NL41531.091.12). All patients signed informed consent.

## Results

Demographics are depicted in Table 1. There were 1322 patients with an ischemic stroke, of which 1043 completed the trigger questionnaire. There were 124 (11.9%) patients with a PFO-associated stroke, of which 83 (66.9%) were closed. The median age of patients with a PFO-associated stroke was 42.1 years (IQR 31.1–53.1) and 45.2% were male. Patients with a bystander PFO and stroke of other etiology (4.6%) had a median age of 43.9 years (IQR 36.0–51.8) and 43.7% were male.

In total, 870 (83.4%) patients underwent TTE, 30 (2.9%) patients underwent TEE and 169 (16.2%) patients underwent both TTE and TEE. Moreover, 143 (13.7%) patients did not undergo echocardiography because the cause of stroke was evident early in the diagnostic process and the demonstration of a PFO would not alter our treatment strategy.

### Trigger factors of PFO-associated stroke

Fever and flu-like disease that occurred within 24 h prior to stroke increased the risk of PFO-associated stroke significantly (RR 26.0, 95% CI 8.0–84.8 and RR 24.2, 95% CI 8.53–68.7, respectively) compared with periods without fever or flu. Furthermore, heavy and severe vigorous physical exercise within 1 h before ischemic stroke increased the risk of having a PFO-associated stroke significantly with an RR of 3.31 (95% CI 2.2–5.1) for all vigorous physical exercise combined (Table 2). Our sensitivity analysis, which excluded all patients who completed the questionnaire 4 weeks or more after their index event, showed an RR increase for fever, flu-like disease, and vigorous exercise (Table 3). No increased risk was found after exposure to caffein-containing coffee consumption, caffein-containing cola consumption, alcohol consumption, cigarette smoking, illicit drug use, or sexual activity.

**Table 2.** Trigger factors for patients with PFO and stroke.

	PFO-associated stroke (*n* = 124)	Exposed^*^ Year (hazard period)	Bystander PFO (*n* = 48)	Exposed^*^ Year (hazard period)
Trigger factor	RR (95% CI)	N (n)	RR (95% CI)	N (n)
Alcohol consumption	0.86 (0.22–3.44)	56 (2)	-	13 (0)
Cigarette smoking	0.74 (0.33–1.68)	26 (14)	0.72 (0.33–1.57)	21 (13)
Coffee consumption	0.92 (0.48–1.77)	89 (13)	0.92 (0.35–2.44)	29 (6)
Cola consumption	2.08 (0.86–5.02)	41 (5)	1.23 (0.12–12.75)	12 (1)
Heavy exercise	3.4 (2.16–5.36)^α^	99 (21)	2.84 (1.05–7.68)^α^	27 (4)
Severe exercise	5.03 (2.59–9.79)^α^	77 (10)	1.72 (0.26–11.53)	18 (1)
Extreme exercise	6.38 (2.82–14.43)^α^	39 (7)	-	10 (0)
Combined vigorous exercise	3.31 (2.16–5.08)^α^	105 (27)	1.76 (0.67–4.60)	28 (4)
Sexual activity	2.0 (0.64–6.20)	87(3)	2.7 (0.37–19.94)	30 (1)
Illicit drug use	-	8 (0)	3.9 (0.28–54.74)	4 (1)
Fever	25.98 (7.96–84.78)^α^	30 (3)	-	12 (0)
Flu-like disease	24.2 (8.53–68.70)^α^	47 (4)	22.75 (3.02–171.33)^α^	14 (1)

PFO: Patent foramen ovale; RR: relative risk, CI: confidence interval.

α RR and 95% CI turned out to be significant.

* The number of patients exposed to the trigger factor during the previous year (the number of patients exposed within the hazard period).

**Table 3.** Sensitivity analysis.

	PFO-associated stroke (*n* = 124)	Excluded patients who filled in questionnaires > 4 weeks after index event (*n* = 74)
Trigger factor	RR (95% CI)	RR (95% CI)
Alcohol	0.86 (0.22–3.44)	0.68 (0.10–4.84)
Cigarette smoking	0.74 (0.33–1.68)	0.54 (0.21–1.42)
Coffee	0.92 (0.48–1.77)	1.15 (0.53–2.48)
Cola	2.08 (0.86–5.02)	2.09 (0.80–5.47)
Heavy exercise	3.4 (2.16–5.36)^α^	3.14 (1.75–5.64)^α^
Severe exercise	5.03 (2.59–9.79)^α^	6.29 (2.35–16.81)^α^
Extreme exercise	6.38 (2.82–14.43)^α^	9.92 (3.47–28.34)^α^
Sexual activity	2 (0.64–6.20)	3.52 (1.12–11.08)^α^
Illicit drug use	-	-
Fever	25.98 (7.96–84.78)^α^	41.96 (12.47–141.20)^α^
Flu-like disease	24.2 (8.53–68.70)^α^	44 (14.75–131.24)^α^

PFO: Patent foramen ovale; RR: relative risk, CI: confidence interval.

α RR and 95% CI turned out to be significant.

## Discussion

We found that vigorous exercise, fever, and flu-like disease may act as a potential trigger factor for PFO-associated stroke. Ekker et al.^7^ elaborate on the influence of trigger factors in distinct non-PFO-associated stroke causes. Given the fact that PFO is a lifelong persisting anatomical variant, our findings may shed light on which (trigger) factors play a role in conversion to a PFO-causing stroke.^27^ There are several biological mechanisms explaining how an infection may convert a PFO into a stroke-causing PFO. First, pathogens can cause an endotheliopathy by direct invasion of the arterial wall when present in the systemic circulation, therefore causing a prothrombotic state.^22^

It is possible that the endocardium of the PFO tunnel is particularly vulnerable for pathogens. Previous research using optical coherence tomography found in situ thrombus formation and abnormal endocardium within the PFO tunnel in PFO-associated stroke patients.^28^

Infection and inflammation are known to be associated with venous thromboembolism (VTE), especially the first 2 weeks after infection onset, gradually declining thereafter.^29^ This may partly explain PFO-associated strokes, as the concept is that a (paradoxical) embolism emerges from the venous circulation, entering the arterial circulation when passing through the PFO.

A possible explanation for the association between vigorous exercise and PFO-associated stroke could be the acute activation of the sympathetic nervous system and therefore increase in heart rate and blood pressure, resulting in increased shear stress.^19^ This may result in platelet deposition, with an accompanying risk of thrombi.^30^ Furthermore, high norepinephrine levels may lead to increased platelet aggregation and oxygen demand.^31^ Hypothetically, increased blood pressure due to exercising could increase the intracardiac pressure and therefore increase the right-to-left shunting. Besides increasing blood pressure, several sports (e.g. weight lifting) could also increase the intrathoracic pressure. Therefore, causing a Valsalva-like maneuver during exercise which is a known risk factor for PFO-associated strokes.^9^

A major strength of this study is the large sample size with ischemic stroke patients based on a tissue-based definition, minimizing the inclusion of stroke mimics. In addition, all echocardiographic images were re-evaluated by an experienced cardiologist. Due to the case-crossover design, patients serve as their own control thereby minimizing the occurrence of confounding bias. All patients with a stroke and PFO who were not found eligible for PFO closure or were included before the instigation of percutaneous closure, were re-evaluated by five independent researchers/ medical doctors (M.H.M.I., M.S.E., N.A.H., E.V., and J.I.V.) to determine if the patient had a PFO-associated stroke.

Our study also has limitations. Recall bias may have played a role as all trigger factors were self-reported by means of a questionnaire. It may be difficult for patients to remember or make an estimated guess how often a trigger occurred in the past year. Several trigger factors could be defined more accurately. For example, we could quantify caffeine consumption in milligrams or number of drinks and separate the different types of exercise instead of using the MET. Furthermore, we have no consecutive data on PFO characteristics, such as diameter or tunnel length. Another limitation of the study is the small number of patients with each of the exposures therefore creating a large confidence interval. Due to the small number of patients exposed to illicit drug use, we were not able to assess whether different types of drugs could serve as a trigger factor for stroke. There could be a bias in reporting, patients may not have been willing to share information about physical activity, the use of drugs or smoking. Of all patients with a PFO-associated stroke, 33.1% did not undergo percutaneous closure. This could (partially) be explained by the fact that a significant number of patients were enrolled prior to the existence of positive RCTs demonstrating the superiority of percutaneous closure over best medical treatment. Unfortunately, there is no available data regarding the reasons for acceptance or rejection of percutaneous closure.

Furthermore, the time window between the event and the trigger may be difficult to define. We tried to minimize recall bias using a sensitivity analysis. A possible solution to address this in future research is to objectify data using health record data and information from wearables. Second, especially in fever and flu-like disease, the width of the 95% confidence interval is quite wide due to the smaller subgroups. Unfortunately, the sample size of patients with an innocent bystander was too small to calculate a reliable RR for fever and possible also for flu-like disease regarding the large confidence interval. A larger cohort of patients with a bystander PFO and a stroke of other etiology are needed to explore the role of trigger factors in these patients. Another limitation in PFO-associated stroke research is the uncertainty about the role of PFO in strokes with competing causes. There is no reliable method to definitively determine which of the competing causes is the actual culprit. We attribute the stroke to the most prevalent cause, considering that most competing etiologies are more likely to cause a stroke than a PFO. Since several patients did not undergo echocardiography due to an evident cause of stroke being identified early in the diagnostic process, the prevalence of patients with a bystander PFO is likely to be much higher than represented in our study. Future research should focus on which patients based on their characteristics, in combination with PFO characteristics, are at the highest risk for PFO-associated stroke. They should also evaluate why most patients with a PFO experience a stroke only once in their life, despite the fact that most trigger factors are present multiple times throughout life. Another interesting question for future research concerns the age aspect of PFO-associated stroke since it occurs more often in young adults, but it is very rare in children, while trigger factors, such as fever and flu-like disease, are abundantly present in childhood. Furthermore, it remains unclear why, for example, labor does not seem to be a major risk factor for PFO-associated stroke, despite the facts that women during childbirth are in a prothrombotic state and need to exert a significant Valsalva maneuver. Finally, imaging studies could detect if patients with a PFO have more “silent infarctions” which lack clinically overt stroke symptoms or if the PFO-associated strokes are indeed sporadic.

## Conclusion

In conclusion, flu-like disease, fever, and vigorous exercise may convert an asymptomatic PFO into a stroke-causing PFO in young adults.

## Supplemental Material

sj-docx-1-wso-10.1177_17474930241242625 – Supplemental material for Trigger factors in patients with a patent foramen ovale—associated stroke: A case-crossover study Supplemental material, sj-docx-1-wso-10.1177_17474930241242625 for Trigger factors in patients with a patent foramen ovale—associated stroke: A case-crossover study by Maikel HM Immens, Merel S Ekker, Esmee Verburgt, Jamie I Verhoeven, Mijntje MI Schellekens, Nina A Hilkens, Esther M Boot, Mayte E Van Alebeek, Paul JAM Brouwers, Renate M Arntz, Gert W Van Dijk, Rob AR Gons, Inge WM Van Uden, Tom den Heijer, Paul LM de Kort, KF de Laat, Anouk GW Van Norden, Sarah E Vermeer, Marian SG Van Zagten, Robert J Van Oostenbrugge, Marieke JH Wermer, Paul J Nederkoorn, Henk Kerkhoff, FA Rooyer, Frank G Van Rooij, Ido R Van den Wijngaard, Catharina JM Klijn, Anil M Tuladhar, Tim JF ten Cate and Frank-Erik de Leeuw in International Journal of Stroke

## References

[1] Hagen PT Scholz DG Edwards WD. Incidence and size of patent foramen ovale during the first 10 decades of life: an autopsy study of 965 normal hearts. Mayo Clin Proc 1984; 59: 17–20. 6694427 10.1016/s0025-6196(12)60336-x

[2] Carroll JD Saver JL Thaler DE , et al. Closure of patent foramen ovale versus medical therapy after cryptogenic stroke. N Engl J Med 2013; 368: 1092–1100. 23514286 10.1056/NEJMoa1301440

[3] Furlan AJ Reisman M Massaro J , et al. Closure or medical therapy for cryptogenic stroke with patent foramen ovale. N Engl J Med 2012; 366: 991–999. 22417252 10.1056/NEJMoa1009639

[4] Vlak MHM Rinkel GJ Greebe P Van der Bom JG Algra A. Trigger factors and their attributable risk for rupture of intracranial aneurysms: a case-crossover study. Stroke 2011; 42: 1878–1882. 21546472 10.1161/STROKEAHA.110.606558

[5] Zhang Z. Case-crossover design and its implementation in R. Ann Transl Med 2016; 4: 341. 27761445 10.21037/atm.2016.05.42 PMC5066041

[6] Koton S Tanne D Bornstein NM Green MS. Triggering risk factors for ischemic stroke: a case-crossover study. Neurology 2004; 63: 2006–2010. 15596741 10.1212/01.wnl.0000145842.25520.a2

[7] Ekker MS Verhoeven JI Rensink KML , et al. Trigger factors for stroke in young adults: a case-crossover study. Neurology 2023; 100: e49–e61. 10.1212/WNL.0000000000201341 36127143

[8] Guiraud V Amor MB Mas J-L Touzé E. Triggers of ischemic stroke: a systematic review. Stroke 2010; 41: 2669–2677. 20947837 10.1161/STROKEAHA.110.597443

[9] Wade E Robinson M Singla A Salerian J. PFO and push-ups: a case series of Valsalva before cryptogenic stroke (5393). AAN Enterprises, 2020, https://www.neurology.org/doi/10.1212/WNL.94.15_supplement.5393

[10] Elgendy AY Saver JL Amin Z , et al. Proposal for updated nomenclature and classification of potential causative mechanism in patent foramen ovale-associated stroke. JAMA Neurol 2020; 77: 878–886. 32282016 10.1001/jamaneurol.2020.0458

[11] Rigatelli G Dell’Avvocata F Cardaioli P , et al. Permanent right-to-left shunt is the key factor in managing patent foramen ovale. J Am Coll Cardiol 2011; 58: 2257–2261. 22078434 10.1016/j.jacc.2011.06.064

[12] Daly S Cassidy T. Thrombus in transit across a patent foramen ovale. N Engl J Med 2023; 389: e45. 10.1056/NEJMicm2208610 37982406

[13] Arntz RM Van Alebeek ME Synhaeve NE , et al. Observational Dutch Young Symptomatic StrokE studY (ODYSSEY): study rationale and protocol of a multicentre prospective cohort study. BMC Neurol 2014; 14: 55. 24655479 10.1186/1471-2377-14-55 PMC3998025

[14] Adams HP Jr Bendixen BH Kappelle LJ , et al. Classification of subtype of acute ischemic stroke. Definitions for use in a multicenter clinical trial. TOAST. Trial of Org 10172 in Acute Stroke Treatment. Stroke 1993; 24: 35–41. 7678184 10.1161/01.str.24.1.35

[15] Immens MHM Van den Hoeven V Van Lith TJ Duijnhouwer TD Ten Cate TJ De Leeuw FE . Heart-Stroke Team: a multidisciplinary assessment of patent foramen ovale-associated stroke. Eur Stroke J 2023; 9: 219–225. 37978872 10.1177/23969873231214862 PMC10916814

[16] Mas J-L Derumeaux G Guillon B , et al. Patent foramen ovale closure or anticoagulation vs. antiplatelets after stroke. N Engl J Med 2017; 377: 1011–1021. 28902593 10.1056/NEJMoa1705915

[17] Saver JL Carroll JD Thaler DE , et al. Long-term outcomes of patent foramen ovale closure or medical therapy after stroke. N Engl J Med 2017; 377: 1022–1032. 28902590 10.1056/NEJMoa1610057

[18] Lee PH Song JK Kim JS , et al. Cryptogenic stroke and high-risk patent foramen ovale: the DEFENSE-PFO trial. J Am Coll Cardiol 2018; 71: 2335–2342. 29544871 10.1016/j.jacc.2018.02.046

[19] Mostofsky E Laier E Levitan EB , et al. Physical activity and onset of acute ischemic stroke: the stroke onset study. Am J Epidemiol 2011; 173: 330–336. 21159732 10.1093/aje/kwq369 PMC4192041

[20] Mostofsky E Schlaug G Mukamal KJ Rosamond WD Mittleman MA. Coffee and acute ischemic stroke onset: the Stroke Onset Study. Neurology 2010; 75: 1583–1588. 20881275 10.1212/WNL.0b013e3181fb443d PMC3120108

[21] Mostofsky E Burger MR Schlaug G Mukamal KJ Rosamond WD Mittleman MA. Alcohol and acute ischemic stroke onset: the stroke onset study. Stroke 2010; 41: 1845–1849. 20634479 10.1161/STROKEAHA.110.580092 PMC2937356

[22] Elkind MSV Boehme AK Smith CJ Meisel A Buckwalter MS. Infection as a stroke risk factor and determinant of outcome after stroke. Stroke 2020; 51: 3156–3168. 32897811 10.1161/STROKEAHA.120.030429 PMC7530056

[23] Lee JP Antin TMJ . How do researchers categorize drugs, and how do drug users categorize them? Contemp Drug Probl 2012; 38: 387–427. 24431475 10.1177/009145091103800304 PMC3888963

[24] Möller J Ahlbom A Hulting J , et al. Sexual activity as a trigger of myocardial infarction. A case-crossover analysis in the Stockholm Heart Epidemiology Programme (SHEEP). Heart 2001; 86: 387–390. 11559674 10.1136/heart.86.4.387 PMC1729949

[25] Kurtz AM Leong J Anand M Dargush AE Shah SA. Effects of caffeinated versus decaffeinated energy shots on blood pressure and heart rate in healthy young volunteers. Pharmacotherapy 2013; 33: 779–786. 23722481 10.1002/phar.1296

[26] Sabbahi A Arena R Kaminsky LA Myers J Phillips SA. Peak blood pressure responses during maximum cardiopulmonary exercise testing: reference standards from FRIEND (Fitness Registry and the Importance of Exercise: a National Database). Hypertension 2018; 71: 229–236. 29255072 10.1161/HYPERTENSIONAHA.117.10116

[27] Kim BJ Kim NY Kang D-W Kim JS Kwon SU. Provoked right-to-left shunt in patent foramen ovale associates with ischemic stroke in posterior circulation. Stroke 2014; 45: 3707–3710. 25358696 10.1161/STROKEAHA.114.007453

[28] Yan C Li H Wang C , et al. Frequency and size of in situ thrombus within patent foramen ovale. Stroke 2023; 54: 1205–1213. 36891906 10.1161/STROKEAHA.122.041524

[29] Schmidt M Horvath-Puho E Thomsen RW Smeeth L Sørensen HT. Acute infections and venous thromboembolism. J Intern Med 2012; 271: 608–618. 22026462 10.1111/j.1365-2796.2011.02473.x PMC3505369

[30] Sakariassen KS Orning L Turitto VT. The impact of blood shear rate on arterial thrombus formation. Future Sci OA 2015; 1: FSO30. 10.4155/fso.15.28 PMC5137878 28031903

[31] Thompson PD Franklin BA Balady GJ , et al. Exercise and acute cardiovascular events: placing the risks into perspective: a scientific statement from the American Heart Association Council on Nutrition, Physical Activity, and Metabolism and the Council on Clinical Cardiology. Circulation 2007; 115: 2358–2368. 17468391 10.1161/CIRCULATIONAHA.107.181485

