# Supplementary material for: Trigger factors in patients with a patent foramen ovale—associated stroke: A case-crossover study
Source: Int J Stroke. 2024 Apr 2;19(7):809–16. doi: 10.1177/17474930241242625 (PMC11298114; doi:10.1177/17474930241242625)
Supplement: sj-docx-1-wso-10.1177_17474930241242625 – Supplemental material for Trigger factors in patients with a patent foramen ovale—associated stroke: A case-crossover study [file sj-docx-1-wso-10.1177_17474930241242625.docx]

**Supplemental Material**

**Trigger factor questionnaire English**

1. **Smoking**
   1. Have you ever smoked?
      - Yes, I still smoke
      - Yes, I have smoked and quit less than 6 months ago
      - Yes, I have smoked and quit more than 6 months ago
      - No, I have never smoked
   2. At what age did you start smoking?

- Age:……..
- I don’t know
  1. How many cigarettes did you smoke on average per day the week before the stroke/ TIA?
- Number:………..
- I don’t know
  1. How many cigarettes did you smoke 1 hour before the onset of the stroke/TIA?
- Number:…………..
- I don’t know

1. **Alcohol**
   1. Do you drink alcohol?
      - Yes
      - No
   2. How many units of alcohol did you consume on average per week in the year before the stroke/TIA?

- Number of units:……………
- I don’t know
  1. How many units of alcohol did you consume the week before the stroke/TIA?
- Number of units:…………..
- I don’t know
  1. How many units of alcohol did you consume 24 hours before the onset of the stroke/TIA?
- Number of units:…………..
- I don’t know
  1. When was the last time you drank alcohol? (number of hours before the stroke/TIA)
- Number of hours:……………
- I don’t know
  1. How many units of alcohol were consumed?
- Number of units:……………
- I don’t know

1. **Drugs**
   1. Have you ever used drugs?

- Yes
- No
  1. Which drugs have you used? (circle what applies)
     1. Cocaine: Yes No
     2. Heroin: Yes No
     3. Methadone: Yes No
     4. Amphetamines: Yes No
     5. Cannabis: Yes No
     6. Hallucinogens: Yes No
     7. Inhalants: Yes No
     8. XTC: Yes No
     9. Anabolic steroids: Yes No
     10. EPO: Yes No
     11. Other, namely:…………………………………………………………………………………………

**If it concerns multiple types of drugs, please answer the following questions about the substance you used the shortest time before the stroke/TIA.**

3.3. How often did you use this drug on average per week in the year before the stroke/TIA?

o Number of times per week: ................

o I don’t know

3.4. How often did you use this drug 1 week before the onset of the stroke/TIA?

o Number of times per week: ................

o I don’t know

3.5. Did you use these drugs 4 hours before the onset of the stroke/TIA?

o Yes

o No

o I don’t know

3.6. When was the last time you used these drugs? (number of hours before the stroke/TIA)

o Number of hours: ...........................

o I don’t know

**4. Coffee**

4.1. Do you ever drink coffee?

o Yes

o No

4.2. How many units of coffee did you consume on average per day in the year before the stroke/TIA? (units per day)

o Number of cups of coffee: ................

o I don’t know

4.3. How many units of coffee did you consume on average per day 1 week before the onset of the stroke/TIA? (units per day)

o Number of cups of coffee: ................

o I don’t know

4.4. How many units of coffee did you consume 1 day before the onset of the stroke/TIA?

o Number of cups of coffee: ................

o I don’t know

4.5. How many units of coffee did you consume 1 hour before the onset of the stroke/TIA?

o Number of cups of coffee: ................

o I don’t know

4.6. When was the last time you drank coffee? (number of hours before the stroke)

o Number of hours before the stroke/TIA: ................

o I don’t know

4.7. How many units were consumed?

o Number of cups of coffee: ................

o I don’t know

**5. Cola**

5.1. Do you ever drink cola?

o Yes

o No

5.2. How many units of cola did you consume on average per week in the year before the stroke/TIA? (units/week)

o Number of glasses of cola: ................

o I don’t know

5.3. How many units of cola did you have 1 week before the onset of the stroke/TIA?

o Number of glasses of cola: ................

o I don’t know

5.4. How many units of cola did you have 1 day before the onset of the stroke/TIA?

o Number of glasses of cola: ................

o I don’t know

5.5. How many units of cola did you have 1 hour before the onset of the stroke/TIA?

o Number of glasses of cola: ................

o I don’t know

5.6. When was the last time you drank cola? (number of hours before the stroke/TIA)

o Number of hours before the stroke/TIA: ................

o I don’t know

5.7. How many units were consumed?

o Number of glasses of cola: ................

o I don’t know

**6. Fever**

6.1. How often did you have a fever in the year before the onset of the stroke/TIA?

o Number of times with fever: ................

o I don’t know

6.2 Did you have a fever 1 week before the onset of the stroke/TIA?

o Yes

o No

o I don’t know

6.3. Did you have a fever 24 hours before the onset of the stroke/TIA?

o Yes

o No

o I don’t know

**7. Flu**

7.1. How often did you have the flu in the year before the onset of the stroke/TIA?

o Number of times: ................

o I don’t know

7.2. Did you have the flu 1 week before the onset of the stroke/TIA?

o Yes

o No

o I don’t know

7.3. Did you have the flu 24 hours before the onset of the stroke/TIA?

o Yes

o No

o I don’t know

**8. Sexual Activity**

8.1. How often per month were you sexually active in the year before the onset of the stroke/TIA?

o Number of times per month: ................

o I don’t know

8.2. Were you sexually active the day before the onset of the stroke/TIA?

o Yes

o No

o I don’t know

8.3. Were you sexually active two hours before the onset of the stroke/TIA?

o Yes

o No

o I don’t know

**9. Exercise**

The following questions are about the number of hours of heavy, very heavy, and extreme exercise you have engaged in during the year, 1 week, and 1 hour before the onset of the stroke/TIA. The table below provides examples of heavy, very heavy, and extreme exercise.

**PLEASE NOTE, you do not need to fill in the example below, please answer the questions on the next page.**

| **MET nr** | **Description** | **Type of activity** | **Number of hours per week** |
| --- | --- | --- | --- |
| 1 | Sleeping | Resting Sunbathing, lying on the couch watching TV | X |
| 2 | Sitting | Reading, desk work, sitting watching TV, highway driving, eating |  |
| 3 | Very light exercise | Office work, city driving, personal care, standing in line, strolling in the park | X |
| 4 | Light exercise | Mopping, slow walking (shopping), bowling, gardening with tools, golfing, sweeping | X |
| 5 | Moderate exercise | Normal walking, golfing, slow biking, downhill skiing, picking up leaves, washing windows, fishing, dancing, painting, wallpapering, light restaurant work | X |
| 6 | Heavy exercise | Jogging, brisk walking, tennis, swimming, cross-country skiing, hoeing, fast biking, heavy household repairs, climbing up and down ladders, ice hockey, softball, bricklaying, heavy restaurant work | X |
| 7 | Very heavy exercise  (lots of sweating and out of breath) | Running, non-stop tennis, pushing car in snow, changing tires, making cement, basketball, climbing ladders with heavy weight | X |
| 8 | Extreme exercise | Extreme exertion Sprinting, uphill running, pushing with all your strength, unusually heavy work or sports | X |

14. How much exercise did you engage in the year before the onset of the stroke/TIA?

a. Average number of hours of heavy exercise per week

o .........................................

b. Average number of hours of very heavy exercise per week

o .........................................

c. Average number of hours of extreme exercise per week

o .........................................

15. How much exercise did you engage in one week before the onset of the stroke/TIA?

a. Average number of hours of heavy exercise per week

o .........................................

b. Average number of hours of very heavy exercise per week

o .........................................

c. Average number of hours of extreme exercise per week

o .........................................

16. How much exercise did you engage in 1 hour before the onset of the stroke/TIA?

a. Number of minutes of heavy exercise

o .........................................

b. Number of minutes of very heavy exercise

o .........................................

c. Number of minutes of extreme exercise

o .........................................
